# The “Mask Effect” of the Emotional Factor in Nurses’ Adaptability to Change: Mental Health in a COVID-19 Setting

**DOI:** 10.3390/healthcare10081457

**Published:** 2022-08-03

**Authors:** José Jesús Gázquez Linares, María del Mar Molero Jurado, María del Carmen Pérez-Fuentes, Ivan Herrera-Peco, África Martos Martínez, Ana Belén Barragán Martín

**Affiliations:** 1 Department of Psychology, Universidad Autónoma de Chile, Providencia 7500000, Chile; jlinares@ual.es; 2 Department of Psychology, Faculty of Psychology, University of Almería, 04120 Almería, Spain; mpf421@ual.es (M.d.C.P.-F.); amm521@ual.es (Á.M.M.); abm410@ual.es (A.B.B.M.); 3 Department of Psychology, Universidad Politécnica y Artística del Paraguay, Asuncion 1628, Paraguay; 4 Nursing Department, Faculty of Health Sciences, Universidad Alfonso X El Sabio, 28691 Madrid, Spain; iherrpec@uax.es

**Keywords:** anxiety, COVID-19, pandemic, emotions, mental health, nurses

## Abstract

During healthcare catastrophes, such as the current COVID-19 pandemic, nurses are exposed to highly stressful situations derived from their work and personal activity. Development of coping strategies for such situations can improve nurses’ physical and mental health. This study analyzed nurses’ adaptability to change, with attention to socio-demographic variables in a COVID-19 setting, and identified the repercussions on their health. This quantitative, observational and cross-sectional study had a sample of 351 nurses aged 22 to 64 with a mean age of 40.91 (SD = 10.98). The instruments used for the study were the ADAPTA-10 questionnaire and the General Health Questionnaire (GHQ-28). It was observed that age, sex, and having a stable partner significantly influenced scores on the emotional, cognitive–behavioral, and adaptation to change factors. Finally, the emotional factor mediated between positive COVID-19 in someone close and the presence of health problems. Understanding the elements that help adapt better to change and adversity enable effective interventions to be developed for improving emotional health of nurses, especially for those in whom there are positive cases of COVID-19 in their personal or work environment.

## 1. Introduction

In December 2019, some cases of pneumonia were found in the city of Wuhan, Hubei Province, China. Although at first their cause was unknown, by the month of January 2020, the pathogen had been identified as a new virus, SARS-CoV-2, which causes the COVID-19 disease [1]. The course of the disease includes a series of systemic physical symptoms such as fever, cough, fatigue, headache, diarrhea, and others, and respiratory affections such as rhinorrhea, pneumonia and acute respiratory distress [1]. In addition, COVID-19 has characteristics that have led to its rapid expansion, with a long incubation period [2] and a high number of asymptomatic carriers [1], which led the World Health Organization to define it as a global pandemic on 31 January 2020 [3]. Added to this are the acute and chronic manifestations of the sequelae of COVID-19, which can be highly complex and multifactorial [4]. Among the factors involved in the development of post-covid sequelae, emotional and social variables could play a relevant role [5]. Thus, for example, in patients with post-COVID-19 chronic pain, anxiety levels and pain intensity largely explain pain sensitization [6].

Due to the rapid propagation of the disease, and the absence to date of effective treatments or vaccines against SARS-CoV-2 [7], healthcare authorities have had to take measures to avoid transmission of the disease and increase in the number of those infected, which has altered people’s daily routines enormously by limiting their movement and interaction with others [8,9]. The measures described and the pandemic itself can have effects not only on physical health caused by the pathogen itself, but also on mental health of the population and of the healthcare professionals, generating situations of continuous stress, anxiety, fear and uncertainty, and others [10,11,12,13].

Nurses during the COVID-19 pandemic, as healthcare professionals in continual contact with patients, are exposed to a very high viral load [14], so as a group they are at an especially high risk of infection [15]. This exposure carries significant risk of affecting their physical health, as shown by the high percentage of healthcare workers infected, as high as 14% in Spain, and 20% in Italy [16].

This risk to nurses’ physical health along with the work overload [17], or the requirement of working with infectious diseases for which they have received no specific training [18], may generate conflict between professional nursing duties and their own personal safety, as well as that of their families. All of this can leave them vulnerable [19,20] to negative effects, such as anxiety, irritability, frustration, fear, and intolerance to uncertainty [21,22], which can provoke alteration in their clinical decision-making capacity, in the quality of care given, and not following work protocols properly [23] due to the tension and stress [17]. This increases the risk of their own infection and of infecting others around them [24]. Additionally, it should not be forgotten that this situation can also affect the mental health of these professionals, as observed in other studies [25].

In this scenario, it should be emphasized that adaptability to change, understood as the personal skill for modifying one’s behavior to adapt to changes occurring around one [26,27], is a competence that nurses must develop to deal with adverse situations. In this sense, resilience can help cope successfully and overcome adverse and stressful situations [28] that generate anxiety in the individual. It should be mentioned that a multitude of factors influence this adaptability [29], some of which are not modifiable, such as sex [30] and age [31]. However, other factors can be worked on to improve individual adaptability [32], such as stress and anxiety management techniques [33,34] or positive coping with surrounding situations [35], among others.

In surroundings with high stress levels, development of coping strategies for such events seems essential. One of the main strategies for such situations is strengthening the emotional dimension of coping [36], which can facilitate adaptability to elements generating stress. Some of the elements that make up this emotional dimension of the capacity for coping are social support and tolerance to uncertainty, depression, and even anxiety [36,37].

Although anxiety is a normal emotion which helps trigger behavior that responds better and more quickly to environmental stimuli, when the duration and intensity of this stimulation become constant, emotional alterations take place that can derive in pathological manifestations, such as behavior disorders, sleep loss, substance abuse, and others [38], which can increase absenteeism [23].

Depression is related to adaptation to change in that it alters the individual’s functional ability, limiting one’s physical and mental activity. With respect to mental activity, the ability to concentrate is diminished, even states of extreme fatigue that impede any activity at all [39,40]. The relationship that some authors, such as Cameron and Schoenfeld [41], have described between depression and continuously high-anxiety situations should be emphasized, because if one cannot cope successfully and finds it impossible to adapt to them, they can lead to a state of depression.

Social support is observed to attenuate the impact that stressful situations can have [27], where both the number of elements in that network as well as support received or empathy perceived buffer their harmful effects [42,43]. Thus, a large, functional professional and personal support network can provide nurses with greater capacity for improving their resilience and positive coping and can redefine potentially stressful situations so that they no longer are [44].

Finally, tolerance to uncertainty could be defined as the set of responses to unknown situations. In this sense, and during the COVID-19 pandemic, not having adequate information or contradictory instructions on procedures generates uncertainty in nurses [45]. Therefore, the relationship between anxiety and uncertainty as generators of emotion alteration is common [46].

Based on the discussion above, the objective of this study was to analyze nurses’ adaptability to change, with attention to sociodemographic variables in a COVID-19 environment, identifying its repercussions on health. The following hypotheses were therefore posed. (H1) The sociodemographic characteristics of nurses do not make significant differences in their adaptability to change. (H2) The presence of COVID-19-positive cases near nurses is negatively related to the emotional factor of adaptability to change. (H3) Adaptability to change correlates negatively with health problems (somatic symptoms, anxiety and insomnia, social dysfunction, and depression). (H4) The emotional factor of adaptability to change works like a mediator in terms of the effect that close positive COVID-19 cases have on the health of nurses.

## 2. Materials and Methods

### 2.1. Participants

Originally, a total of 505 nursing professionals gave their consent to participate in the study. After review, 154 cases were discarded because 148 of the questionnaires were unfinished and 6 contained incoherent answers to the control questions distributed at random throughout the questionnaire.

Thus, the study sample was made up of 351 nurses, all of them residents of Spain, aged 22 to 64, and with a mean age of 40.91 (SD = 10.98). Of the total sample, 86% (*n* = 302) were women. The distribution of marital status was 61.3% (*n* = 215) married or with a stable partner, 32.5% (*n* = 114) single, 5.1% (*n* = 18) were separated or divorced, and the remaining 1.1% (*n* ≥ 4) were widowed.

Of the participants, 9.1% (*n* = 32) had been diagnosed as COVID-19 positive. The participants were also asked about the existence of positive cases near to them, to which 62.7% answered affirmatively (*n* = 220).

### 2.2. Instruments

An ad hoc questionnaire was used to collect participant sociodemographic data and ask them questions related to COVID-19 (impact on income, positive diagnosis of COVID-19 or not, and any close COVID-19 positive cases).

The Cuestionario de Adaptación al Cambio (Adaptability to Change Questionnaire) (ADAPTA-10) [36] consists of 10 items answered on a five-point Likert-type scale. It provides a total score on adaptability to change and scores on two factors: an emotional factor related to anxiety and distress that could appear under change, and a cognitive–behavioral factor related to the ability to control, manage, and act in different situations. In this case, reliability was ω = 0.81 and GLB = 0.90 for the total scale, ω = 0.83 and GLB = 0.86 for the emotional factor and ω = 0.76 and GLB = 0.81 for the cognitive–behavioral factor.

The General Health Questionnaire (GHQ-28) [47], i.e., the Spanish adaptation validated by Lobo et al. [48], contains 28 items with four answer choices and provides information on somatic symptoms, anxiety and insomnia, social dysfunction, and depression. Of the correction methods possible, the Likert scale was used, scoring each answer from 0 to 3. The instrument’s reliability is ω = 0.93 on the complete scale, and for each of the subscales: somatic symptoms (ω = 0.86), anxiety and insomnia (ω = 0.90), social dysfunction (ω = 0.81), and depression (ω = 0.91).

### 2.3. Procedure

Data were collected using a CAWI (Computer Aided Web Interviewing) survey, which facilitated its diffusion on social networks and instant messaging among groups of nurses. Data were collected in the seventh and eighth weeks of confinement in Spain, that is, from 1–12 May 2020.

Participation was voluntary and before starting to answer the questionnaire, information about the study and its purpose was given on the first page. They were also asked to answer sincerely, assuring the anonymity of their answers. The participants provided their informed consent by marking a box for the purpose, which then gave them access to the questionnaire. To detect random or incongruent answers, control questions were inserted throughout the questionnaire. The study was approved by the University of Almería Bioethics Committee (Ref: UALBIO2020/021).

### 2.4. Data Analysis

First, the *t*-test for independent samples was applied to find out whether there were any differences in adaptability to change, and Cohen’s d [49] to quantify the effect size. In addition, associations between variables were explored with Pearson coefficient correlation analyses. Then mediation analyses were performed, taking as the predictor close COVID-19 positive cases, as mediators the adaptability to change factors, and as result variables, the health subscales (somatic symptoms, anxiety/insomnia, social dysfunction, and depression). For this, the jAMM module for advanced mediation models was used [50]. Instrument reliability was estimated using the McDonald’s omega, following Ventura-León and Caycho [51]. The Greatest Lower Bound (GLB) was also calculated.

## 3. Results

### 3.1. Nurses’ Adaptability to Change: Socio-Demographic Variables and COVID-19 Environment

In the first place, age had no significant association with the emotional factor (r = 0.009; *p* = 0.868; 95% CI −0.09, 0.11), the cognitive–behavioral factor (r = 0.07; *p* = 0.172; 95% CI −0.03, 0.17), or the total score on adaptability to change (r = 0.04; *p* = 0.442; 95% CI −0.06, 0.14).

In addition, when the mean scores of men and women were compared, no statistically significant difference was observed in any of the adaptability measures: emotional factor (t(349) = 1.86, *p* = 0.063), cognitive–behavioral factor (t(349) = −0.52, *p* = 0.602), and total adaptability scale (t(349) = 1.12, *p* = 0.262). Even though there were no significant differences, the men’s mean score on the emotional factor (M = 15.93, SD = 4.22) was slightly higher than women’s (M = 14.73, SD = 4.19), while for the cognitive–behavioral factor, women (M = 20.11, SD = 2.58) had higher mean scores than the men (M = 19.89, SD = 3.41).

By sentimental situation (without/with partner) at the time data were collected, no statistically significant differences were found for the emotional factor (t(349) = −0.27, *p* = 0.787), the cognitive–behavioral factor (t(349) = −1.32, *p* = 0.187), or the total adaptability scale (t(349) = −0.82, *p* = 0.410). Although there were no significant differences, those who had a stable partner had higher mean scores (emotional factor: M = 14.94, SD = 4.12; cognitive–behavioral Factor: M = 20.23, SD = 2.51; Total: M = 35.18, SD = 5.45) than those who did not have a stable partner (emotional factor: M = 14.82, SD = 4.37; cognitive–behavioral factor: M = 19.84, SD = 2.98; Total: M = 34.66, SD = 6.11).

**Figure 1:**
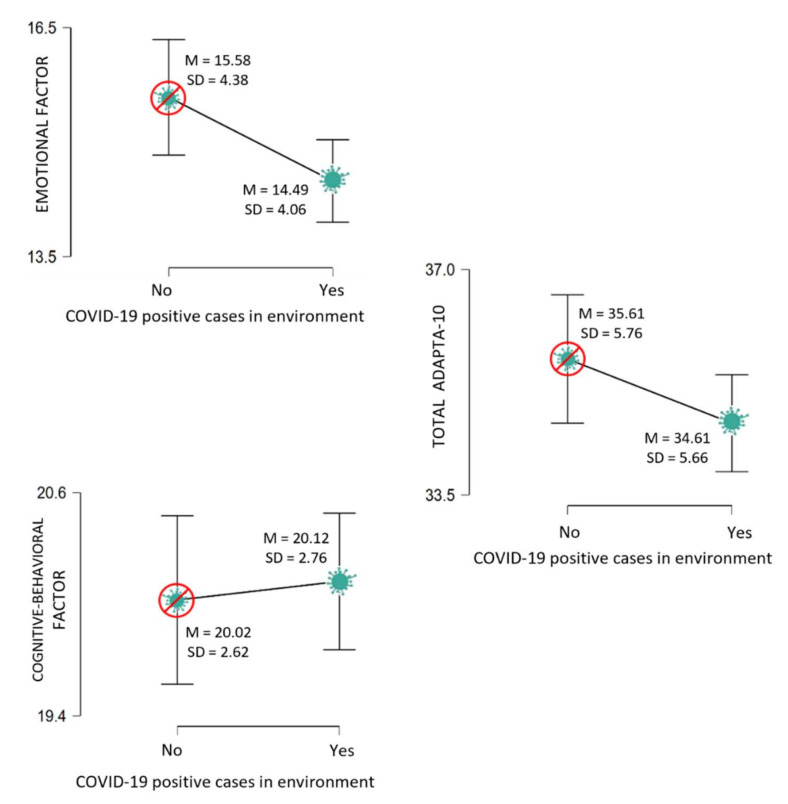
Adaptability to change by presence of positive close COVID-19 cases. Descriptive plots.

Moreover, there were no significant differences in adaptability to change among professionals who had been diagnosed with COVID-19 and those who had not. However, there were statistically significant differences by presence or not of close positive cases (Figure 1) in the emotional factor of adaptability to change, where in this case, those who stated having a close positive case had a lower mean score.

### 3.2. Nurses’ Adaptability to Change and Its Relationship with Health

Table 1 shows the correlation matrix between the ADAPTA-10 Adaptability to Change questionnaire factors and total score and the various GHQ-28 subscales. The two factors (emotional and cognitive–behavioral) and the total score on adaptability to change were negatively correlated with health problems: somatic symptoms, anxiety/insomnia, social dysfunction, and depression.

**Figure 2:**
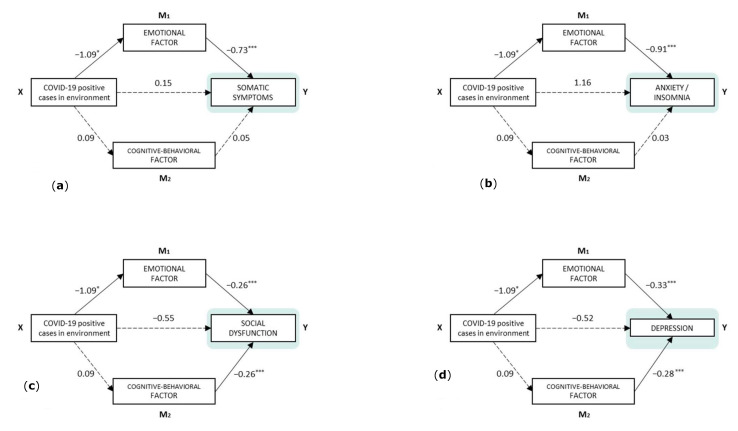
Components of direct effects and model paths. (**a**) Y = Somatic symptoms, (**b**) Y = Anxiety/insomnia, (**c**) Y = Social dysfunction, (**d**) Y = Depression. Note. * *p* < 0.05, *** *p* < 0.001 (Model diagram notes: categorical independent variables (factors) are shown with only one rectangle, but their effect is estimated using contrast variables: “COVID-19 positive cases in environment” = no (−0.5)/yes (0.5)).

Several mediation analyses were performed to check the mediating role of the two adaptability to change factors. In all cases, the predictor was the presence of a close COVID-19 positive case, and as output variables each of the GHQ-28 dimensions. Figure 2a–d show these models.

As observed, the existence of close positive cases of COVID-19 had no significant direct effect on the presence of health problems. As indirect effects (Table 2), the emotional factor of adaptability to change was a significant mediator between close COVID-19 cases and the four health subscales: somatic symptoms, anxiety/insomnia, social dysfunction, and depression. The cognitive–behavioral factor, however, did not act as a mediator in the relationship with close positive cases and health problems in any case. 

Finally, the total effect was significant in the model that took anxiety/insomnia as the dependent variable. In the model that predicts somatic symptoms, the *p*-value for the total effect was tendential. 

## 4. Discussion

This study analyzed the emotional and cognitive–behavioral dimensions of nurses’ adaptability to change under the threat represented by the COVID-19 pandemic itself, as well as actions carried out to manage it.

In the first place, it should be emphasized that the results confirmed that the sociodemographic data related to gender did not influence perception of the risk of COVID-19 at all, nor were there any significant differences between the cognitive–behavioral and emotional factors of adaptability to change. This is contrary to what has been observed in the general population, where men have higher adaptability to change in adverse situations and less perception of vulnerability to threat [30]. In the sample of nurses analyzed, age was not observed to effect adaptability to change either, or to have any greater effect on the emotional or cognitive–behavioral factors, such as a more consolidated social support network, as was observed in the general population, where older people adapted better to change [1,31,42]. Having a stable partner also offered results that showed significant differences in the emotional, and cognitive-–behavioral factors and in the adaptability to change scale; however, the data collected allowed a certain trend to be inferred for the emotional factor to positively influence adaptability to change in adverse events for those with a stable partner, in agreement with other authors who have found that having a stable partner increases one’s perception of wellbeing [52] and provides emotional and social support [53] even in situations such as the pandemic.

In the second place, coinciding with our second hypothesis, it was observed that the perception of risk, adaptability to change, and emotional and cognitive–behavioral factors were not affected by the participant having been diagnosed with COVID-19 or not. However, having received news of a positive diagnosis in the family was associated with low emotional factor scores. This may be because of nurses’ concern about spreading the disease to their families [24], which is related to the appearance of high vulnerability [19], and such negative effects as anxiety, frustration, and fear, making the nurse less adaptable to changes in the workplace [21,22].

In the third place, high scores in adaptability to change, as well as on the emotional and cognitive–behavioral factors were shown to enable nurses to cope with the appearance of negative effects on health, such as insomnia or depression. This could be explained by a relationship between a high level of anxiety and the appearance of alterations in the individual [28]. Therefore, a better ability to manage anxiety would reduce the appearance of alterations derived from it, such as depression [41] or alteration in social behavior patterns [32].

Finally, this study showed that the emotional factor, made up of elements such as social support, tolerance to uncertainty, depression, and anxiety management, was a mediator between close COVID-19 cases and the appearance of nurses’ somatic symptoms, anxiety/insomnia, social dysfunction, and depression. COVID-19 positive cases close to nurses caused uncertainty [17,54] and fear of infecting their loved ones [8] as stressful elements generating anxiety and facilitating the appearance of fears [25]. Clear examples are feelings of uncertainty and guilt of not knowing whether one is a carrier of the virus or not or of whether they have protected themselves well enough not to be infected or whether they have infected their loved ones [18].

The situations of fear and uncertainty described [17,54] are associated with the existence of continuously high stress [45]. Anxiety also commonly appears in situations of uncertainty, for example, not knowing if the COVID-19-positive cases close to nurses could have been their fault, which generates strong anxiety in them, deriving emotional alterations [46]. Furthermore, this anxiety, derived from such situations, can lead to somatic symptoms such as sleep disorders, headaches, distress, weakness, difficulty interacting with the social environment, and others [23,38]. Previous studies have reported a direct association between the existence of high-anxiety environments, in which, due to the uncertainty, it is impossible to adapt to them to reduce the anxiety, with the appearance of depression [39,41].

Another of the elements that make up the emotional facet of adaptability to change and that is affected by the appearance of a close COVID-19 positive case is related to social support. As the family is one of the essential elements within that social support [27], the doubt that it may have been the nurse that caused the contagion [18] makes them not reach out to that network for fear of rejection [46], and not receive the benefit of the buffer effect of a social network in stressful situations [42,44].

In spite of the possible positive repercussions of the findings shown in this study, it had some limitations mainly related to the study design, which was cross-sectional. These data should be completed with longitudinal studies that enable causal relationships to be established between the variables studied. This is especially necessary in the study of burnout as a syndrome that depends on multiple factors such as length of service and other factors such as gender. In this line, it should also be mentioned that most of the sample was made up of women, although this reflects the reality of the nursing profession in Spain. Finally, this study is limited insofar as the sample was small and only nurses, which makes it difficult to extrapolate the conclusions to other healthcare professions. In the sample, there were more women than men. We did analyze gender differences, but when the mean scores of men and women were compared, no statistically significant difference was observed in any of the adaptability measures.

## 5. Conclusions

The main conclusion of this study was that there is an important relationship between the elements of the emotional factor of adaptability to change and the impact of having COVID-19 cases close to them on nurses personally and at work.

In this situation, we think that the improvement of skills that increase their capacity for resilience are shown to be essential within adaptability of nurses to highly demanding environments and in those where the situations that generate stress around them are constant. Likewise, and in a situation such as the appearance of a case of COVID-19 in their family, nurses need to have the emotional tools necessary for the somatic symptoms associated with constantly high levels of anxiety not to appear, affecting their personal and professional life.

It would be essential for healthcare institutions and organizations to propose and implement a series of psychosocial intervention programs developing personal and technical competences focused on effective reduction of stress and anxiety or strengthening emotional management. Such skills and competences could be considered protective factors against situations that generate uncertainty for these professionals, who carry out their professional and personal activities in direct contact with patients [55]. Spanish nurses would therefore be better prepared for situations that require high demand, such as the COVID-19 pandemic or any other type of catastrophe that could take place.

The findings in this study are highly relevant because of the impact of the COVID-19 pandemic, and the care plans and actions implemented for it, has had on nurses around the world. This situation has made nursing environments very demanding and generated a high level of personal and work anxiety. This can affect clinical practice and nursing care, generating disaffection, not following protocols, and by lowering their quality, even patient recovery.

Care of nurses’ emotional health therefore seems to be a priority for any healthcare system, since they are the professionals who are in closest contact with the patients and have high responsibility for their care and recovery. Having predictive models that enable the effect of stressful situations on nurses’ health to be known would enable the most sensitive professionals to be detected and design strategies for their prevention, manage them better, and improve care quality.

## Figures and Tables

**Table 1:** Adaptability to change and health. Pearson correlations.

		GHQ_SS		GHQ_AI		GHQ_SD		GHQ_D	
Emotional factor	Pearson’s r	−0.630	***	−0.762	***	−0.412	***	−0.474	***
	*p*-value	<0.001		<0.001		<0.001		<0.001	
	Upper 95% CI	−0.562		−0.714		−0.322		−0.389	
	Lower 95% CI	−0.689		−0.803		−0.496		−0.551	
Cognitive–Behavioral factor	Pearson’s r	−0.180	***	−0.235	***	−0.342	***	−0.360	***
	*p*-value	<0.001		<0.001		<0.001		<0.001	
	Upper 95% CI	−0.076		−0.133		−0.246		−0.265	
	Lower 95% CI	−0.279		−0.331		−0.431		−0.448	
Total ADAPTA-10	Pearson’s r	−0.550	***	−0.673	***	−0.466	***	−0.520	***
	*p*-value	<0.001		<0.001		<0.001		<0.001	
	Upper 95% CI	−0.472		−0.611		−0.380		−0.439	
	Lower 95% CI	−0.619		−0.727		−0.544		−0.592	

Notes: GHQ-SS = Somatic symptoms, GHQ-AI = Anxiety/insomnia, GHQ-SD = Social dysfunction, GHQ-D = Depression. *** *p* < 0.001.

**Table 2:** Indirect and total effects 95% CI.

				95% CI				
Type	Effect	Estimate	SE	Lower	Upper	β	z	*p*
Indirect	COVID19 ⇒ EM Factor ⇒ GHQ-SS	0.806	0.341	0.134	1.471	0.080	2.353	0.019
	COVID19 ⇒ CB Factor ⇒ GHQ-SS	0.005	0.018	−0.030	0.042	5.72 × 10^−4^	0.307	0.759
	COVID19 ⇒ EM Factor ⇒ GHQ-AI	0.999	0.422	0.171	1.826	0.096	2.367	0.018
	COVID19 ⇒ CB Factor ⇒ GHQ-AI	0.003	0.012	−0.021	0.028	3.49 × 10^−4^	0.288	0.773
	COVID19 ⇒ EM Factor ⇒ GHQ-SD	0.290	0.128	0.039	0.541	0.045	2.265	0.024
	COVID19 ⇒ CB Factor ⇒ GHQ-SD	−0.026	0.079	−0.182	0.129	−0.004	−0.334	0.739
	COVID19 ⇒ EM Factor ⇒ GHQ-D	0.364	0.158	0.054	0.675	0.053	2.301	0.021
	COVID19 ⇒ CB Factor ⇒ GHQ-D	−0.028	0.084	−0.193	0.137	−0.004	−0.334	0.739
Total	COVID19 ⇒ GHQ-SS	0.964	0.530	−0.074	2.004	0.096	1.820	0.069
	COVID19 ⇒ GHQ-AI	1.167	0.5491	0.0914	2.2437	0.1129	2.127	0.033
	COVID19 ⇒ GHQ-SD	−0.289	0.352	−0.981	0.402	−0.043	−0.820	0.412
	COVID19 ⇒ GHQ-D	−0.184	0.377	−0.923	0.555	−0.026	−0.488	0.625

Note. COVID19 = COVID-19 positive cases in environment, EM = Emotional factor, CB = Cognitive–Behavioral factor, GHQ-SS = Somatic symptoms, GHQ-AI = Anxiety/insomnia, GHQ-SD = Social dysfunction, GHQ-D = Depression (Confidence intervals computed with Delta method).

## Data Availability

All data generated or analysed during this study are included in this published article.
